# Supplementary material for: Detection of carbapenem-resistant Klebsiella pneumoniae on the basis of matrix-assisted laser desorption ionization time-of-flight mass spectrometry by using supervised machine learning approach
Source: PLoS One. 2020 Feb 6;15(2):e0228459. doi: 10.1371/journal.pone.0228459 (PMC7004327; doi:10.1371/journal.pone.0228459)

Original gel images contained in the manuscript’s Figure 3.

M: The 1-kb DNA ladder.

X: Lanes not included in the manuscript’s Fig. 3.

S: The strain #36 was used as the standard strain and included in every experiment as a control.

Date of each run was shown above.

KODAK 1D Image Analysis Software (v. 3.6) was used to capture the images.

20180302

M 36 X X 18 19 20 X

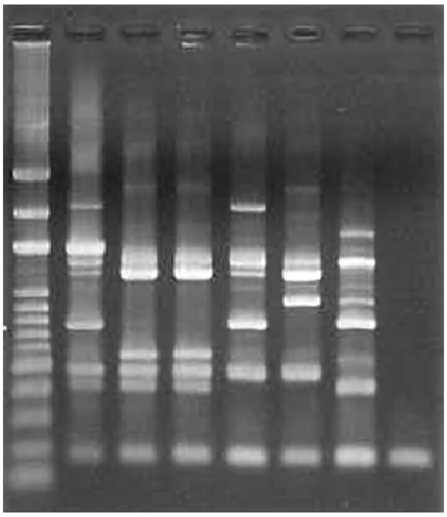

20180306

M S 5 8 9 11 X 2 3 4 21 X 17 31 40 X X

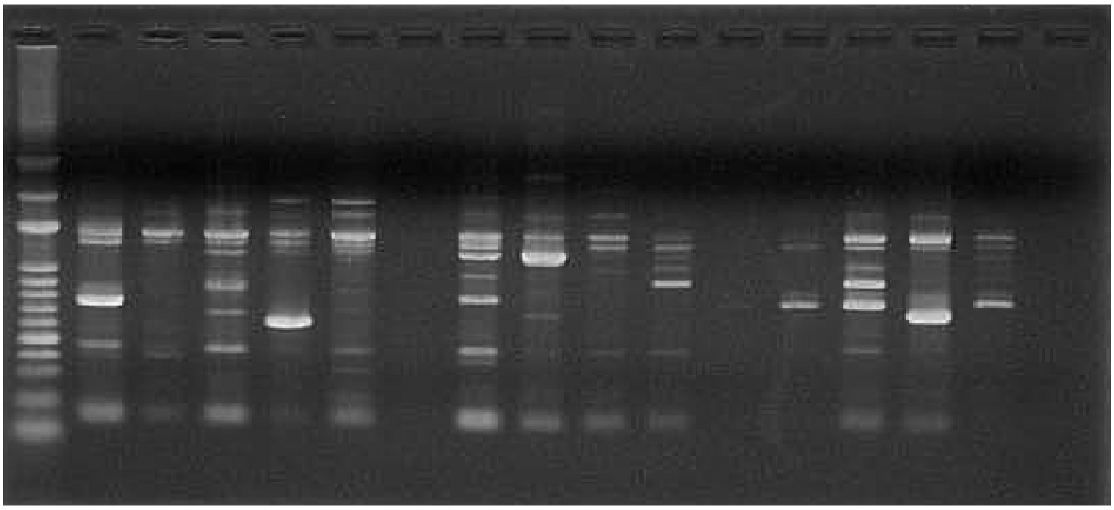

20180312

M S 1 7 10 13 14 15 X X X X 24 26 29 16 22

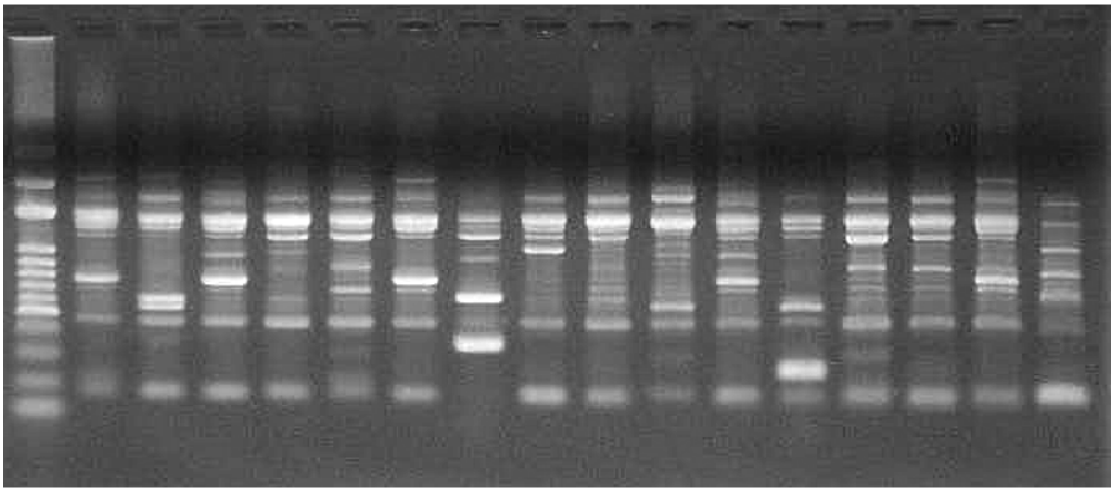

20180314

M S X X X 23 25 27 X X X 30 X X X 28 X

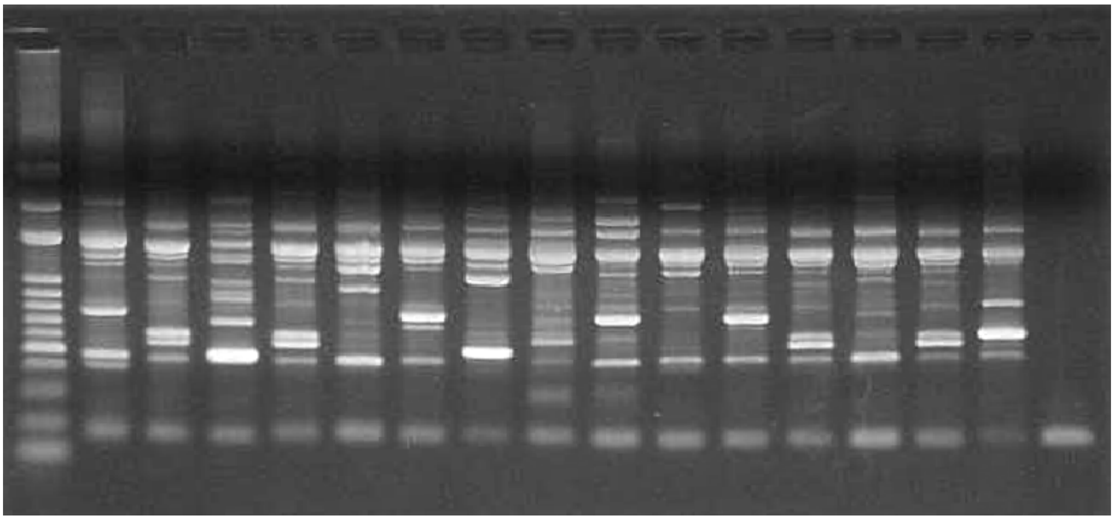

20180319

M S X X 33 X X X X 41 X X X X X X X

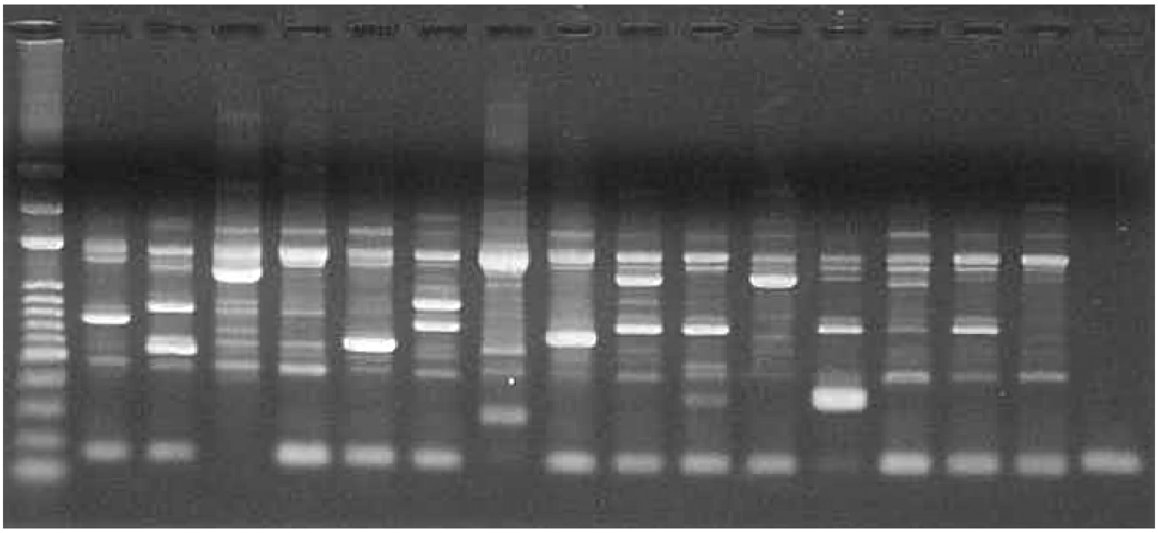

20180321

M S 39 X X X X X

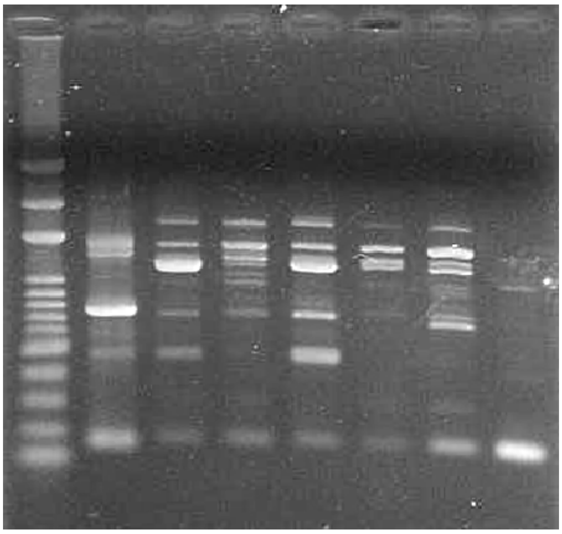

20180322

M S 12 32 34 37 35 38 42 43 45 44 46 X X X X

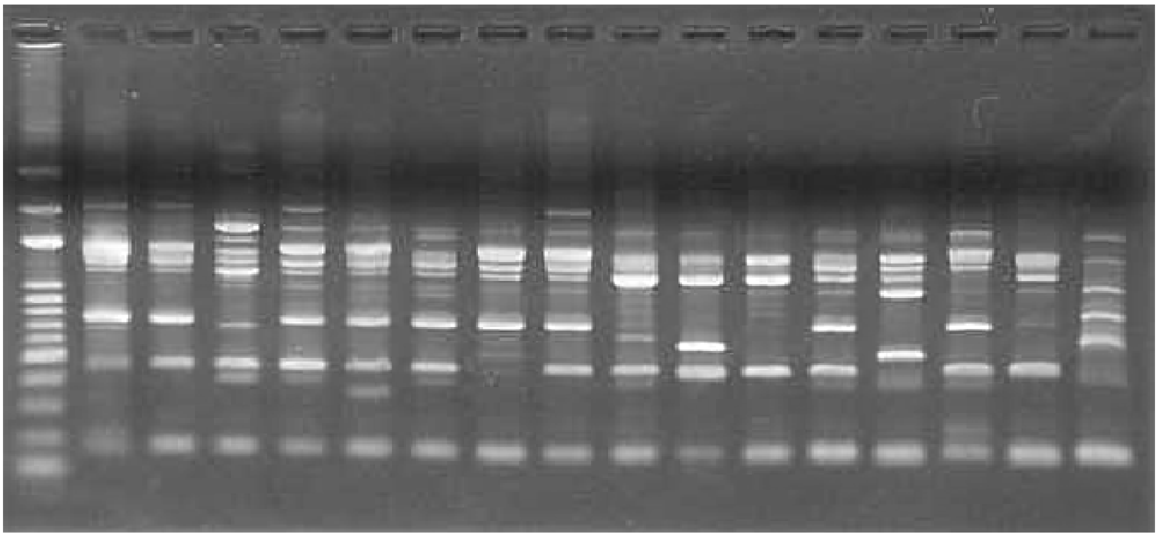

20180419

M S X 6 X X X X X X X X X X X X X

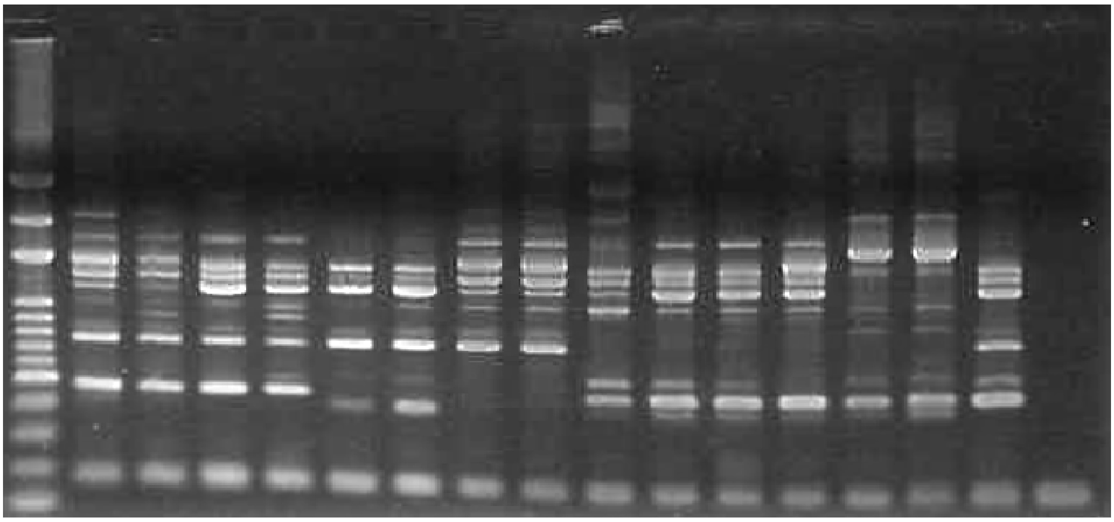

Supplement: S1 Fig — (PDF) [file pone.0228459.s001.pdf]
