# Supplementary material for: Detection of carbapenem-resistant Klebsiella pneumoniae on the basis of matrix-assisted laser desorption ionization time-of-flight mass spectrometry by using supervised machine learning approach
Source: PLoS One. 2020 Feb 6;15(2):e0228459. doi: 10.1371/journal.pone.0228459 (PMC7004327; doi:10.1371/journal.pone.0228459)
Supplement: Attachment — Submitted filename: Response to reviewers-20191225R1.doc [file pone.0228459.s002.doc]

**Response to Editor**

To the Editor

Plos ONE Editorial Office

Re: PONE-D-19-26377

Thank you for your constructive review of this manuscript. My co-authors appreciate the opportunity to address the reviewer's comments and to revise the manuscript accordingly.

**Reviewer #1**

Major comments:

1. The conclusion in the abstract section was rewritten.
2. The introduction was revised to state the problem more clearly and explain why it is important and why we chose the study method. We also made a brief review of the previous studies, including what these approaches do in terms of drug resistant bacteria, the techniques they used, and how these approaches compares to the presented work.
3. Figure 2 and discussions in the manuscript on that topic was removed, as suggested by the reviewer.
4. *Klebsiella* spp. are intrinsically resistant to penicillins, i.e. all of the CRKP as well as CSKP are penicillin resistant. Therefore, the peaks that differentiate between CRKP and CSKP are not related to penicillins, since both CRKP and CSKP are resistant to penicillin.
5. It is not able to find potential peptides/proteins of interest from just m/z ratio alone. We demonstrated that CRKPs can be differentiated from CSKPs through supervised machine learning method. It is possible to consider the specific attributes (such as m/z ratio) as biomarkers for the defined classes. Whether these target peaks are potential biomarkers require further study which analyze and process large series of bacterial strains (Line 296-298).
6. Feature selection using t-test was performed using Mathematica software v. 12 (Wolfram Research, Champaign, IL) (Line 173-174). Both t-test and PCA essentially reduce the dimensionality of the data, however, the overall performance by using PCA was inferior to t-test (accuracy was 85-92%, with 74%-83% of sensitivity and 92%-100% of specificity).

Minor comments:

1. The sentence was corrected as “a significant pathogen causing healthcare-associated infections”. (Line 26).
2. The full name of the acronyms at its first appearance were added: ToF (Line 27) CDC WHO (Line 49-50) PCR (Line 70) MIC (Line 146-147) CHCA (Line 157) RF (Line 109).
3. The abstract was rewritten.
4. Full name of CSKP Deleted.
5. The urgency of carbapenem resistance as a threat to healthcare was described in Introduction (Line 52-66).
6. This sentence was corrected as “Carbapenem resistance can be mediated by production of carbapenemases or by the combination of outer membrane porin expression disruption and production of various β-lactamases” (Line 50-52). The role of β-lactamase inhibitors combined with β-lactam antibiotic to address the resistance was added. (Line 88-89).
7. The full name of KPC was added. (Line 55)
8. The epidemiology of carbapenem-producing *K. pneumoniae* was described in Introduction (Line 55-63).
9. We rewrote the paragraph to indicate why spectral differences between resistant and sensitive isolates are expected (Line 78-97).
10. The first three approaches usually detect molecules beyond the detection limit of the MALDI-TOF instruments now commonly employed in clinical microbiology were explained more clearly (Line 78-100).
11. Solvent extraction involves several washing and centrifugation steps and is more labor-intensive than direct analysis of bacteria without additional protein extraction, which is usually done in routine work flow (Line 116-118).
12. SVM abbreviation was used.
13. The carbapenemase phenotype in the Vitek2 AES in our hospital predicts imipenem and ertapenem susceptibility, but not meropenem. Meropenem was not included in our panel (Line 145-149).
14. Sample size was specified as approximately three millimeters in diameter, according to the User Manual VITEK MS Clinical Use-Workflow) (Line 154).
15. All the procedures and reagents were conducted according to the manufacture’s recommendations for identification of bacteria in a routine laboratory without any modification.
16. The Reference of *E. coli* as reference strain was cited (Line 161).
17. The score 90% is a common practice. When isolates were re-analyzed, the scores from the repeats only were used in the analysis, not averaged. N=1 was used throughout this study (Line 164).
18. No isotopes or salts were used in the spectral analysis.
19. The data transferred from the Vitek MS acquisition station was analyzed by Vitek MS RUO v4.12 (Line 167).
20. In this study, each peak was treated as an independent observation (Line 183). It was not necessary to apply any corrections for the multiple student t-testing to adjust the individual *p* value for each peak to keep the overall error rate to less than or equal to the user-specified *p* cutoff value.
21. RAPD procedures were revised (Line 207-216).
22. Bacteria isolates included in this study were isolated from clinical specimens including 7 (7.4%) pus/wound, 5 (5.3%)sputum, 20 (21.1%) urine, 54 (56.8%) blood, and 5 (5.3%) body fluids like ascitic fluid, bile, pleural fluid and 4 (4.2%) other specimens like catheter tips.
23. This study was approved by the Institutional Review Board (VGHKS18-CT8-07) (Line 143-144).
24. Dimensionality is the number of peaks and is count data.
25. The conclusion that Random forest algorithm outperformed than the other algorithm was based on the ability to correct classification. Comments discussing the repeatedly misclassified isolate are written in discussion (Line 320-324). The L1O cross validation procedure was used to assess the predictive ability of different learning algorithms. The tested data is not part of the training set used in the building model and served as a blinded sample (Line 312-319).
26. These three differentially expressed peaks needs to be analyzed and processed using a large series of bacterial strains in the future to see if they are potential biomarkers (Line 296-298).
27. We used all the five machine learning algorithms that were offered in the Mathematica software v. 12 (Wolfram Research, Champaign, IL).
28. Figures and Tables were cited in the text.
29. The training set and testing set was described in Materials and method (Line 178-179).
30. The X and Y axis in Figure 3 were specified.
31. Reference formatting was corrected.

**Reviewer #2**

1. The corresponding author is a statistics expert and performed analysis and interpretation.

The revised manuscript is attached. We hope the revised manuscript is in satisfactory form and we look forward to hearing from you again soon.

Sincerely yours,

Fu-Chuen Chang

Professor

Department of Applied Mathematics

National Sun Yat-sen University

70 Lien-hai Rd.

Kaohsiung 804, Taiwan

(O) 886-7-525-2000-3823

Fax 886-7-525-3809
